# Zoonotic *Dirofilaria* sp. “hongkongensis” in subcutaneous nodules from dogs and cats, Hong Kong SAR

**DOI:** 10.1186/s13071-024-06544-7

**Published:** 2024-11-15

**Authors:** Thamali Manathunga, May Tse, Livia Perles, Frederic Beugnet, Vanessa Barrs, Domenico Otranto

**Affiliations:** 1 Department of Veterinary Clinical Sciences, Jockey Club College of Veterinary Medicine and Life Sciences, City University of Hong Kong, Kowloon Tong, Hong Kong SAR China; 2 Department of Veterinary Medicine, University of Bari Aldo Moro, Bari, Italy; 3 Boehringer-Ingelheim Animal Health, Lyon, France

**Keywords:** Subcutaneous dirofilariosis, *Dirofilaria* sp. “hongkongensis”, Dogs, Cats, Zoonoses

## Abstract

**Background:**

*Dirofilaria* sp. “hongkongensis” is a putative *Dirofilaria* species, initially identified in subcutaneous nodules in humans in Hong Kong and in other South and Southeast Asian regions. While it differs genetically from the better-known zoonotic species, *Dirofilaria repens* and *Dirofilaria immitis*, information on the lesions caused by *Dirofilaria* sp. “hongkongensis” in the hosts as well as on its biology is scarce. This study documents for the first time the presence of this filarioid nematode in subcutaneous nodules in dogs and cats in Hong Kong, where it was originally described in human patients, therefore providing evidence for the zoonotic nature of this parasite.

**Methods:**

Records of Veterinary Diagnostic Laboratory of City University of Hong Kong were searched between 2019 and 2024 for histological reports of possible filarioid-associated lesions. Tissue samples were collected by excisional surgical biopsy and processed with routine paraffin techniques. Selected slides were stained using various staining techniques [i.e., hematoxylin and eosin, periodic acid–Schiff (PAS), Grocott methenamine silver (GMS) or Ziehl–Neelsen (ZN) and Gram stain]. DNA from formalin-fixed paraffin-embedded tissue were extracted, submitted to conventional polymerase chain reaction (cPCR) and sequencing (i.e., *cox*1 and *12S* rRNA genes) and phylogenetic analyzed.

**Results:**

A total of five subcutaneous nodules from four cats and one from a dog with histopathology suggestive of filariosis were selected. The presence of *Dirofilaria* sp. “hongkongensis” was morphologically and molecularly confirmed in one dog and one cat. Both histopathological presentation and phylogenetic analysis enabled classification of this species close to *D. repens* and within the subgenus Nochtiella. In the remaining three cases, one showed histological evidence of aberrant nematode migration, while non-parasitic causes were identified in the other two.

**Conclusions:**

This study provides the first evidence of *Dirofilaria* sp. “hongkongensis” in subcutaneous nodules in cats and dogs. The histology of clinical lesions of this filarioid species herein described is closely related to those caused by *D. repens*. Overall, this species should be considered in differential diagnoses of subcutaneous lesions in both animals and humans in the region.

**Graphical Abstract:**

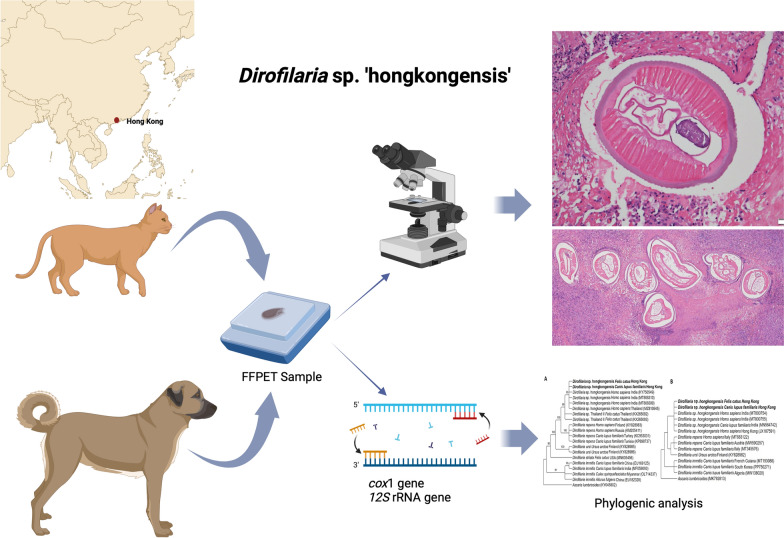

## Background

Dirofilarioses are mosquito-borne parasitic diseases that mainly affect canines, felines, and other mammals. Of the 27 species included in the genus *Dirofilaria*, the most important are *Dirofilaria immitis* and *Dirofilaria repens*, which are prevalent in dogs and may cause human infection [1, 2]. While *D. immitis* has a worldwide distribution, *D. repens* is primarily endemic in Europe, Africa, and Asia [3, 4]. The occurrence of canine dirofilariosis is affected by the density of competent Culicinae vectors (e.g., *Aedes albopictus, Culex pipiens, Aedes aegypti, Culex quinquefasciatus*) and the presence of dogs and other susceptible hosts that maintain endemicity of the infection [5–7]. Unlike *D. immitis*, which causes a severe and potentially fatal condition in dogs (i.e., canine heartworm disease), *D. repens* usually leads to subcutaneous infections, which are often subclinical [8]. Infections of both *Dirofilaria* spp. are diagnosed through the observation or molecular detection of microfilariae (mf) in blood [9]. Other diagnostic tests are represented by the detection of antigens for *D. immitis*, or by the removal of the nematode from subcutaneous nodules for *D. repens* [9, 10]. For the latter species, infection in dogs causes skin swelling, hyperpigmentation, subcutaneous granulomas containing adult or immature worms and local pruritus [8, 11], although subclinical (asymptomatic) forms are also prevalent [6].

Human dirofilariasis is mainly caused by *D. repens* in the Old World and by *D. immitis* in the New World [2]. Among cases reviewed worldwide, *D. repens* was identified in 72.2%, followed by *D. immitis* (6.9%) and other *Dirofilaria* species. [1]. Importantly, *D. repens* is increasingly recognized as a filarioid of zoonotic concern in Europe and Asia [6], with human infections mostly associated with subcutaneous (i.e., 50.17%) and ocular filariasis (i.e., 22.2%) [12–14], and less commonly with pulmonary lesions (i.e., 13.02%) [1].

Both species of *Dirofilaria* are endemic in China and Southeast Asia (SEA) [15]. Epidemiological surveys on *D. immitis* conducted in China revealed an overall prevalence of 13.18% in dogs with wide regional variations, ranging from 1.1% in northwest regions to 22.8% in the southwest [15]. In addition, seroprevalence of *D. immitis* observed in feline populations in China ranged from 1.93% to 4.5% on the basis of antigen detection [16–18]. In contrast, in SEA a 3.5% overall prevalence of *D. immitis* was recorded in dogs, with the highest rates reported from the Philippines, Taiwan, and Malaysia (i.e., 17.9%, 8.3%, and 6.7%, respectively) [19]. Other studies also reported the presence of *D. immitis* in cats in Thailand (i.e., 36.4%) [20, 21], and Indonesia (i.e., 1.3%) [19] on the basis of molecular analysis and antigen detection, respectively. Information about *D. repens* in China is limited to a few case reports in humans of subcutaneous and ocular dirofilariasis, with no data available for dogs and cats [15, 22, 23]. Cases of human subcutaneous and ocular dirofilariasis caused by *D. repens* have been reported in SEA, including Vietnam [24, 25], Malaysia [26, 27], and Thailand [28, 29]. In addition, a high genetic diversity of *Dirofilaria* species within the Nocthiella subgenus is recorded in SEA [2] and the existence of putative species, such as *Dirofilaria* sp. “hongkongensis,” *Dirofilaria* sp. “Thailand II,” and *Dirofilaria* sp. “Thailand III,” has been the subject of debate [30, 31].

*Dirofilaria* sp. “hongkongensis” was first defined as a molecular taxonomic unit in 2012, from nematodes collected in three human patients presenting with subcutaneous nodules and in blood samples from stray dogs in the Hong Kong Special Administrative Region (SAR) of China [14]. This putative species was subsequently identified in human patients with a similar clinical presentation in India and Thailand [32–34] as well as in Europe, after a history of traveling to India [35, 36]. Despite its molecular characterization as a putatively valid species, the proposed scientific name '*Candidatus Dirofilaria* hongkongensis' remains a *nomen nudum* due to lack of a holotype and of a proper morphological description, as per the International Code of Zoological Nomenclature [37]. Nonetheless, genetic analysis suggests that it may be a cryptic species of *D. repens* [30]. Although human dirofilariasis has been documented in Hong Kong and attributed to *D. repens* and *Dirofilaria* sp. “hongkongensis” in ocular [38] and subcutaneous forms [39], there is a notable knowledge gap regarding the prevalence and the clinical presentation of subcutaneous dirofilariosis in canines and felines. Therefore, in the present study we attempt to identify the *Dirofilaria* species responsible for subcutaneous nodules in dogs and cats in the Hong Kong SAR and describe associated nodular lesions.

## Methods

### Animals and sampling procedure

Records of the laboratory information management system (LIMS) at the Veterinary Diagnostic Laboratory of City University of Hong Kong between 2019 and 2024 were searched for histological reports of possible filarioid-associated lesions using keywords (“dog”, “cat”, or “canine” or “feline”, and “eosinophilic”, and “granulomatous” or “pyogranulomatous” and “heartworm” or “dirofilaria” or “parasite” or “endoparasite”). In total, four cats and one dog with suspicious subcutaneous nodules presenting at different anatomic locations were identified (Table 1). Diagnostic submission data included the location and diameter of each subcutaneous nodule. Tissue samples were collected by excisional surgical biopsy fixed in 10% neutral-buffered formalin, processed with routine paraffin techniques, and 5 µm thick sections were stained with hematoxylin and eosin. Selected slides were stained with periodic acid–Schiff (PAS) and/or Grocott methenamine silver (GMS) for fungi, or Ziehl–Neelsen (ZN) for mycobacteria, and Gram stain for bacteria to rule out non-parasitic etiologies.

**Table 1:** Clinical history and signalment of animals examined, along with anatomical site, general presentation, and size of subcutaneous nodules

Sample ID	Sample collection month (year)	Animal	Breed	Age	Sex (reproductive status)	Clinical history	Nodular location (size)
1	August (2019)	Cat	Domestic shorthair	3 years and 6 months	Male Neutered	Weight loss and vomiting. A mass was observed in the right scrotum 8 weeks previously	Right scrotal sac (12 × 10 × 5 mm)
2	December (2023)	Cat	Domestic shorthair	8 months	Male Neutered	Mammary mass with progressive enlargement	Mammary mass adjacent to third right nipple (9 × 25 × 29 mm)
3	January (2024)	Cat	Domestic shorthair	4 years	Male Neutered	Right inguinal and popliteal lymph node enlargement	Right inguinal (35 × 21 × 18 mm) and popliteal lymph node (4 × 12 × 30 mm)
4	February (2024)	Cat	Domestic shorthair	6 months	Male	A chronic nonhealing wound in the perianal area was managed as an open wound. Multidrug-resistant *Escherichia coli* and *Enterococcus faecalis* were isolated in culture from the wound	Perianal area (seven pieces of tissue from 5 × 5 × 6 to 7 × 10 × 30 mm)
5	August (2020)	Dog	Mongrel/mixed breed	7 years and 7 months	Female	Left-sided mammary swelling	Left mammary swelling (140 mm long and 70 mm diameter)

### DNA extraction and molecular screening

Extraction of DNA from formalin-fixed paraffin-embedded tissue (FFPET) samples was performed using the EZ2® Connect system (Qiagen GmbH, Hilden, Germany) with EZ1 and EZ2 DNA tissue kit (Qiagen GmbH, Hilden, Germany), according to the manufacturer’s protocol. Conventional polymerase chain reaction (cPCR) was performed targeting a portion of mitochondrial cytochrome oxidase c subunit 1 (*cox*1) gene using primers Diro-cox1-F (5′-GCTTTGTCTTTTTGGTTTACTTTT-3′) and Diro-cox1-R (5′-TCAAACCTCCAATAGTAAAAAGAA-3′) [33] and *12S* ribosomal RNA (*12S* rRNA) gene using primers 12S nem F (5′-GTTCCAGAATAATCGGCTA-3′) and 12S nem R (5′-CTACCATACTACAACTTACGC-3′) [40], following the protocol previously described. All cPCR products were analyzed by electrophoresis on 2% agarose gel, purified and sequenced. Sequences were edited using Geneious Prime® 2024.0.3 and compared with the complete mitochondrial genome of *Dirofilaria* sp. “hongkongensis” (Genbank AN: NC_031365).

For phylogenetic inference, sequences from the present study were aligned with those retrieved from GenBank using MAFFT software version 7 [41]. The best evolutionary model was chosen under the Akaike information criterion (AIC) and Bayesian (BI) phylogenetic analyses were performed using CIPRES gateway (available at https://www.phylo.org/). Markov chain Monte Carlo (MCMC) simulations were run for 10^6^ generations with a sampling frequency of every 100 generations and a burn-in of 25. The phylogenetic tree edition and rooting (outgroup) were performed using TreeGraph 2.0 beta software [42].

## Results

**Fig. 1:**
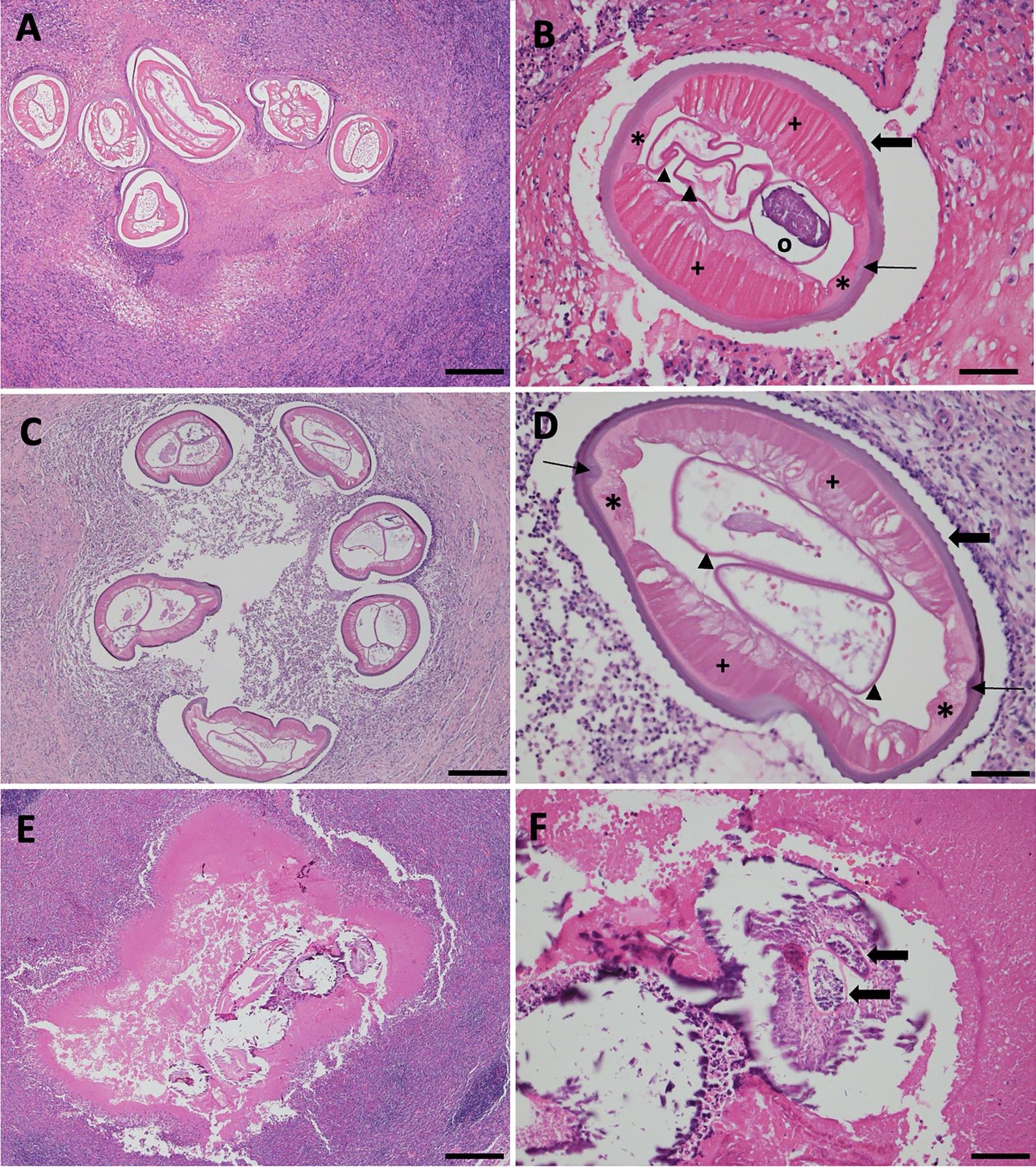
Histologic sections of nodules from case 1 and 5 (hematoxylin and eosin staining). Mass at the right scrotum of a cat with tangential sections of nematode, embedded in the core of inflammatory cells. Scale bar 200 µm. (**A**). At higher magnification the nematode cuticle is evenly spaced with longitudinal ridges (thick arrow), lateral cords (*), internal ridges (thin arrow), tall coelomyarian-polymyarian musculature (+), ovary (black circle), and uteri (arrow heads) (**B**). Mass at the mammary gland of a dog with multiple cross sections of nematode. Scale bar 200 µm. (**C**). At higher magnification, longitudinal ridges (arrow), lateral cords (*), internal ridges (thin arrow), tall coelomyarian-polymyarian musculature (+), and reproductive tract (arrow head) is evident. Scale bar 50 µm. (**D**). Mammary mass in a cat. Scale bar 200 µm. (**E**) At higher magnification parasite fragments show structures resembling the possible reproductive tract of a nematode (arrows). (**F**). Scale bar 50 µm

Chronic granulomatous to pyogranulomatous inflammation with marked eosinophilia was observed in all five cases. Visible cross sections of nematodes were noted in two cases; one in a scrotal wall mass in a cat (case 1) (Fig. 1A) and the other in a mammary mass in a dog (case 5) (Fig. 1C). In both cases, histological sections revealed multiple cross and tangential sections of nematodes approximately 200 µm in diameter, surrounded by peripheral eosinophil-rich inflammatory infiltrates including macrophages and lymphocytes admixed with cellular debris (Fig. 1A, C). In case 1 (Fig. 1A, C), the nematodes measured 240 µm to 420 µm in width, while in case 5 (Fig. 1B, D), they ranged from 360 µm to 460 µm. In cross-sections of nematodes, the cuticle displayed approximately 80–110, low, smoothly rounded, evenly spaced external longitudinal ridges, ranging from 2 µm to 11 µm apart (Fig. 1B, D). The thickness of the cuticle ranged from 5 µm to 9 µm. At higher magnification, beneath the cuticle, a clear hypodermis was observed, followed by well-developed, tall polymyarian-type musculature, which was interrupted by two lateral chords (Fig. 1B, D). An ovary and uterine tubules (Fig. 1B), as well as uterine tubules (Fig. 1D), were seen in the center of the cross section of nematode (Fig. 1B, D). One of the remaining three cases (case 2) showed histological evidence suggestive of aberrant nematode migration with dense infiltrate of neutrophils, eosinophils, macrophages, and lymphocytes and liquefactive necrotic debris scattered with fragments of a degenerated parasite (Fig. 1E, F). Finally, in the other two cases non-parasitic causes were identified (i.e., Gram negative coccobacilli in case 3 and oomycosis or zygomycosis with faint characteristic hyphae on GMS stain in case 4). However, these cases (cases 2, 3, and 4) showed a chronological evolution of the infection with histological evidence of nematode presence, characterized by peripheral eosinophilic infiltrates, necrotic cores with laminated keratin spikes, and structures resembling mineralized parasites. Though eosinophilic oval structures indicative of residual parasite remnants and the formation of granulation tissue were observed, molecular analysis did not allow detection of genomic DNA of *Dirofilaria* species.

In total, two out of the five FFPET samples (cases 1 and 5) that tested positive for both the *cox*1 and *12S* rRNA gene PCR assays were identified as *Dirofilaria* sp. “hongkongensis” through Sanger sequencing. Nucleotide sequences of the partial *cox*1 gene were deposited in the GenBank sequence database (accession numbers PQ327004 and PQ327005), as were the *12S* rRNA sequences (accession numbers PQ032750 and PQ032751). For *cox*1, cases 1 and 5 had 100% and 99.85% nucleotide identity, respectively, with a complete mitochondrial genome sequence of *Dirofilaria* sp. “hongkongensis” isolated from a human eyelid in India (Genbank accession number NC_031365). For the *12S* rRNA sequences, both cases had 100% nucleotide identity to the same GenBank sequence (NC_031365).

**Fig. 2:**
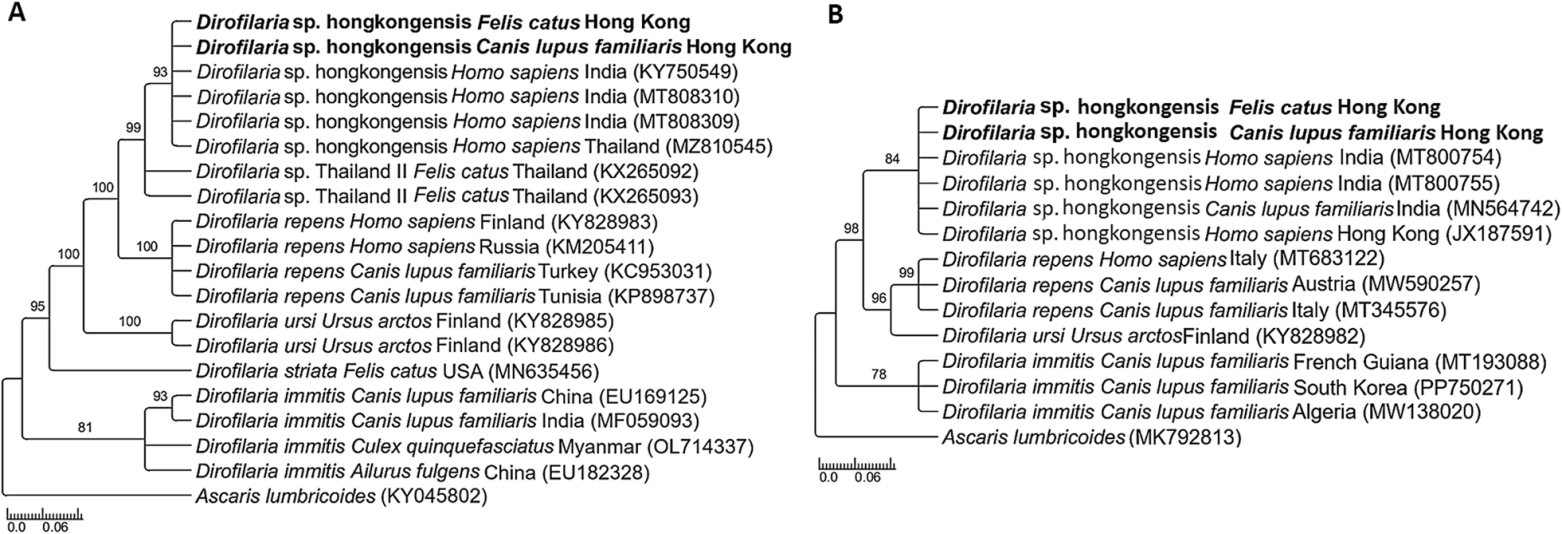
Clades of *Dirofilaria* species were based on *12S* rRNA gene (**A**) (accession numbers PQ032750–PQ032751) and *cox*1 gene (**B**) (accession numbers PQ327004–PQ327005). The phylogenetic trees were inferred using Bayesian inference. The sequences are aligned for the respective genes and included information on the host of collection, geographical provenience, and accession number. Sequences from the present study are highlighted in bold. *Ascaris lumbricoides* was used as outgroup and numbers at nodes correspond to the posterior probability support values

On the phylogenetic analysis of the *12S* rDNA sequences, the sequences from our two Hong Kong cases clustered within the clade of *Dirofilaria* sp. “hongkongensis” sequences from India (GenBank accession no. KY750549, MT808310, MT808309) and Thailand (GenBank accession no. MZ810545) (Fig. 2A). The phylogenetic analysis based on the *cox*1 gene demonstrated that our sequences clustered with *Dirofilaria* sp. “hongkongensis” from India (accession no. MT800754, MT80755, MN564742) and Hong Kong (accession no. JX187591) (Fig. 2B).

## Discussion

Following the description of subcutaneous dirofilariasis caused by *Dirofilaria* sp. “hongkongensis” in human patients from Hong Kong, here we documented for the first time the occurrence of this filarioid in subcutaneous nodules in cats and dogs from the same region, thus reinforcing the zoonotic nature of this scarcely known parasitosis. The clinical and histological picture of presented cases was similar to that of *D. repens*, which is the primary cause of zoonotic dirofilariosis in Europe. Histopathological analysis of the cases in our study identified distinct external longitudinal cuticular ridges, which are characteristic feature of the Nochtiella subgenus [43].

In the case of canine dirofilariosis caused by *D. repens*, the nematode typically resides in the subcutaneous tissues, where it can migrate freely and cause nodular lesions [8]. The etiopathogenesis of *D. repens*-associated subcutaneous filarial nodules is unclear, especially whether they originate from direct mechanical action induced by the nematode, the chronic inflammaory response exerted by the infected dog [8], or from a combination of both. Overall, only 12% of dogs with subcutaneous dirofilariosis caused by *D. repens* present with cutaneous nodules [44]. The subcutaneous nodules caused by *Dirofilaria* sp. “hongkongensis” herein described were located in a posterior body region (scrotal and mammary regions), as reported in 85% of dermatological lesions by *D. repens* (i.e., lumbosacral region, hind limbs, and perianal area) [44]. The posterior localization and subcutaneous tissue preference observed in both species raise the question of whether the pathogenicity of *Dirofilaria* sp. “hongkongensis” is similar to that of *D. repens*. Additionally, clinical signs such as anorexia and vomiting were observed in 35% and 26% of previously reported cases of *D. repens* with dermatological manifestations in dogs, respectively. However, these dogs were co-infected with other vector-borne diseases, with babesiosis being the predominant infection [44, 45]. In the current study, case 1 had a history of anorexia and vomiting while concurrent infections were not reported. Conversely, both positive samples were collected in August, during the summer, which provides a favorable environment for mosquito vectors [5].

On the basis of the histopathology of our two cases, the pattern of inflammation caused by *Dirofilaria* sp. “hongkongensis” was characterized by cross sections of nematodes surrounded by a mix of inflammatory cells including neutrophils, macrophages, and lymphocytes with a marked eosinophil infiltrate. This pattern is typical in foreign body inflammation, and was also seen in the three other PCR-negative cases in our study. The histopathological findings associated with the *Dirofilaria* sp. “hongkongensis” nodules in our study are similar to that of *D. repens* [46].

In our study, PCR and sequencing of the partial sequences of the mitochondrial *cox*1 and *12S* rRNA genes genes enabled molecular identification of the species of filarioid associated with canine and feline subcutaneous nodules. Phylogenetic analysis of the obtained *cox*1 and *12S* rDNA sequences revealed a close relationship with *Dirofilaria* sp. “hongkongensis” isolated from human cases. This finding highlights the zoonotic potential of this species, making it a parasite to consider in diagnosis and prevalence studies in the region. Additionally, the molecular identification and phylogenetic analysis of *Dirofilaria* sp. “hongkongensis” provides significant insights into its classification and evolutionary history. On the basis of its morphology and phylogenesis, we confirm its phylogenetic relationship with *D. repens*, and place it within the Nochtiella subgenus [30, 32–36, 40].

## Conclusions

The present study provides the first documented evidence of *Dirofilaria* sp. “hongkongensis” in subcutaneous nodules in cats and dogs, with close phylogenetic relationship to nematodes of the same species from humans, highlighting its zoonotic potential. On the basis of these findings, we suggest that dirofilariosis should be considered in the differential diagnosis of subcutaneous nodules of dogs and cats as well as in humans.

## Data Availability

All data generated or analysed during this study are included in this published article and can be accessed at NCBI—GenBank—Nucleotide platform (https://www.ncbi.nlm.nih.gov/genbank/).
